# *Stryphnodendron* Species Known as “Barbatimão”: A Comprehensive Report

**DOI:** 10.3390/molecules23040910

**Published:** 2018-04-15

**Authors:** Tatiana M. Souza-Moreira, Geisiany M. Queiroz-Fernandes, Rosemeire C. L. R. Pietro

**Affiliations:** 1 Department of Drugs and Medicines, School of Pharmaceutical Sciences, Sao Paulo State University-UNESP, Rodovia Araraquara-Jaú, km 1, Araraquara 14800-903, Sao Paulo, Brazil; souzatm@gmail.com; 2 Pró Reitoria de Pesquisa e Pós-graduação, Universidade do Sagrado Coração, Bauru 17011-160, São Paulo, Brazil; geisiany.queiroz@usc.br

**Keywords:** ethnopharmacology, medicinal plant, biological activity, tannin, catechin, wound healing

## Abstract

*Stryphnodendron* spp., popularly known as “barbatimão”, is the native Brazilian tree most often employed to treat wounds and infections. The aim of the present study was to highlight the importance of *S. adstringens,* as well as other *Stryphnodendron* species recognized as “barbatimão”, to human health, depicting the relevance of ethnopharmacological knowledge to scientific evidence for uses, related chemical compounds, development of pharmaceutical formulations, and the establishment of toxicity parameters. For this purpose, the literature databases PubMed, Scielo, Lilacs, CAPES Thesis and Google Scholar were searched until 2017. It was observed that stem bark was the primary part of the plant used, mainly as a decoction, for wound healing and treatment of infectious and inflammatory disorders. Confirmed biological activities, including wound healing, anti-inflammatory, antioxidant, and antimicrobial activities, were related to the presence of compounds from tannin class, mostly proanthocyanidins. Toxicity parameters for stem bark were inconclusive, but toxicity was observed to a significant extent when seeds were ingested by cattle or other animals. Due to these important and confirmed biological activities, government policy encourages the phytotherapic use of *S. adstringens*, and some formulations with stem bark extracts were developed and patented. Furthermore, antiprotozoal, hypoglycemic and antiviral activities were identified as promising.

## 1. Introduction

Plants are a source of molecules with a wide variety of applications, and humanity has learned to harness their benefits and to recognize their toxic effects throughout history. Ethnopharmacological uses of plants represent part of each culture around the world, so it is no wonder that isolated active molecules are being used in standard preparations [1,2]. Today, natural drugs, including plants and their derivatives, are one of the biggest sources of approved medicines [2]. Brazil has extensive biodiversity, and many exotic species have been introduced, reflecting a rich folk medicine with the influence of native, African, and European peoples [3,4]. In that sense, government policies were established over the years to protect Brazilian biodiversity and to stimulate drug development and phytotherapy in the country’s health system [5,6,7].

In Brazil, one of the native trees commonly used is known as “barbatimão”, from which stem barks are prepared as decoctions or infusions with the main purpose of wound and infection healing [8,9]. On the other hand, the broad beans are also recognized as an abortive for cattle when they are eaten in the field [10,11]. Other popular names of these trees include “barbatimão-verdadeiro”, “barba-de-timão”, “chorãozinho-roxo” and “casca-da-virgindade”. The scientific name of the species is *Stryphnodendron adstringens* (Mart.) Coville, with accepted synonyms *Acacia adstringens* Mart., *Mimosa barbadetimam* Vell., *Mimosa virginalis* Arruda, *Stryphnodendron barbatimam* Mart., and *Stryphnodendron barbadetiman* (Vell.) Mart. [12,13]. However, other *Stryphnodendron* species are also popularly known as “barbatimão” as *S. obovatum* Benth. (synonym of *S. rotundifolium* Mart.), *S. polyphyllum* Mart., and *S. rotundifolium* Mart.. Other species such as *Abarema cochliocarpos* (Gomes) Barneby & Grimes, *Pithecellobium cocliocarpum* (Gomez) Macbr., and *Dimorphandra mollis* Benth. can be mistakenly referred in the literature as “barbatimão” but they are recognized as “falso-barbatimão” which could be freely translated as “fake-barbatimão”. There are 42 species in the *Stryphnodendron* genus presently, and they are disseminated in the Neotropical region, from Costa Rica in Central America to the south of Brazil, with the majority of species in Brazil present either in the rainforest or in the Brazilian savanna (“Cerrado”) [14]. Phylogenetic analysis of the *Stryphnodendron* genus showed very short internal branches but clustered together savanna species as *S. adstringens* and *S. rotundifolium*, whereas species from the rainforest such as *S. polyphyllum* formed a different cluster, indicating rapid evolution and morphological diversification in the group. *Abarema*, *Pithecellobium* and *Dimorphandra* were not included in this study as their genera were not closely related to *Stryphnodendron* [15].

Thus, data compilation reviewing the studies already done with *Stryphnodendron* spp. barks will contribute to the phytotherapeutic use of “barbatimão” in the health care system and to the development of efficient formulations. In addition, this data compilation will also highlight the gaps still present in literature in order to elucidate the safe use of the barks in a sustainable way and to create a future in which the active molecules of the extract can be utilized as new drugs or new drug prototypes. Furthermore, by stimulating the conservation of the species and disseminating its benefits to the scientific community, more drugs can be developed for treatment of wounds as well as infectious and inflammatory disorders.

In that sense, the aim of the present work is to review the existing information available about the benefits of *S. adstringens* and other “barbatimão” species from the *Stryphnodendron* genus to human health, thereby demonstrating the relevance of folk knowledge to scientifically-proven biological activities, formulations developed, toxicity parameters, and the main compounds involved.

## 2. Data Collection Methodology

The scientific names of the *Stryphnodendron* genus referred to as “barbatimão” were selected for search in databases. Those names comprised *S. adstringens*, *A. adstringens*, *M. barbadetimam*, *M. virginalis*, *S. barbatimam*, *S. barbadetiman*, *S. discolor*, *S. obovatum*, *S. polyphyllum*, and *S. rotundifolium*. Searches were performed in the following databases: Pubmed (https://www.ncbi.nlm.nih.gov/pubmed/), Scielo (http://www.scielo.br/), Lilacs (http://lilacs.bvsalud.org/), and the CAPES Brazilian Thesis databank (http://bancodeteses.capes.gov.br/banco-teses/#!/) up to 5 November, 2017. Complementary information was searched in Google (http://www.google.com).

## 3. General Aspects on Literature about *Stryphnodendron*

**Figure 1:**
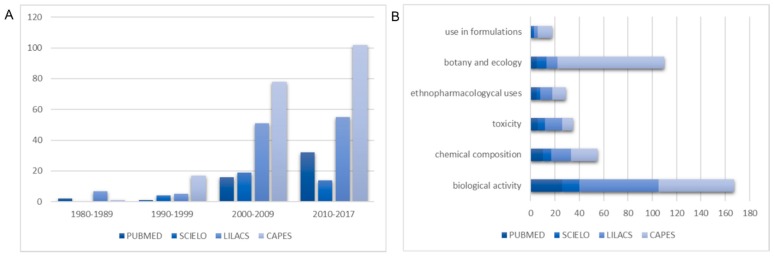
Number of publications in the searched databases according year (**A**) and research area (**B**).

A survey of the databases PubMed, Scielo and Lilacs identified about 200 papers covering “barbatimão” species (*S. adstringens*, *S. obovatum*, *S. polyphyllum* and *S. rotundifolium*, including synonyms), and more than 200 Master and PhD Theses in Brazil were found in CAPES. After the year 2000, the number of publications about *Stryphnodendron* spp. known as “barbatimão” increased about 10-fold and twice in journals indexed in PubMed after 2010 (Figure 1A). Most of the publications were related to the biological properties of the species, mainly for the treatment of internal and external infectious and inflammatory diseases, but also for wound and ulcerative wound healing related to the astringent and cicatrization properties, highlighting the ethnopharmacological attributes of the species. Demonstrating the ethnopharmacological use of a plant requires well-conducted scientific studies correlating the plant results with a well-established standard [1]. Attention should be payed to studies of biological activity with inconclusive data, which, while not confirming ethnopharmacological use, can be a step in the right direction [16]. To that end, the Brazilian Pharmacopeia and Government have amassed the efforts of recognized scientific institutions and internationally published papers to enable proper identification of “barbatimão” species, their active chemical constituents, and their therapeutic uses according to the proven efficacy of the species [17,18,19].

Chemical composition and standardization of methods to analyze the barks constitution are expressive. Although there are some studies related to plant toxicity of “barbatimão” species for cattle, there are few studies focusing on toxicity levels and safety in humans. Different medicinal topical products derived from bark extracts are being developed, and their activity has been demonstrated (Figure 1B).

## 4. Botanical Features and Sustainable Management Aspects

Plants from genus *Stryphnodendron* belongs to the Fabaceae Lindl. family, which comprises more than 200 genera [12]. Trees of the genus are small to medium sized evergreens, with an unbranched trunk, and the stem generally has tortuous and thick rust-colored bark [13,20]. *S. adstringens* trees have a low and round top, while *S. polyphyllum* has a large top, and the top of *S. rotundifolium* is round and diffuse [20]. 

*S. polyphyllum* has the shortest petiole at 3–6.5 cm, which contrasts with *S. adstringens* at 6.5–9 cm and *S. rotundifolium* at 2.5–10 cm. Species are more easily differentiated by their bipinnate compound leaves; *S. adstringens* has 5–7 pairs of leaflets in opposite insertion with 5–6 pairs of second order leaflets alternately inserted, limb is asymmetrical ovoid, sometimes elliptical, with 1.5–3.5 × 1–2.5 cm emarginated or round apex, flat margin narrowly thickening; card form limb, glabrous, concolor, visible veins immersed in the limb in the superior face and salient in the inferior face. On the other hand, leaflets of *S. polyphyllum* shows higher number of pairs of leaflets (11–18, although less are present in leaflets close to the branch apex) in opposite insertion, also with higher number of pairs of second order leaflets (14–23) and insertion subopposite; limb is slightly asymmetrical, oblong (sometimes distal pairs are obovate and rarely proximal pairs are elliptical to oval), with 3–8 × 1.5–4 mm, mostly round apex but, sometimes, apex is obtuse, margin is revolute; card form limb, pubescent, subconcolor, darker superior face with invisible veins immersed in the limb and slightly visible in the inferior face [20,21,22]. *S. rotundifolium* has 6–13 pairs of leaflets in opposite insertion in distal pairs and subopposite in proximal pairs, 5–12 pairs of second order leaflets with alternate insertion (except distal pairs have opposite insertion), asymmetric to symmetric limb, generally orbicular, ovoid, elliptical or elliptical-ovoid (distal pairs are obovate) with 7–18 × 6–13 mm, asymmetrical apex generally retuse to emarginate, but sometimes round, sub-revolute margin, slightly thickening; card form limb, darker superior face with slightly visible immersed veins and central vein sometimes ridged, which is salient in the inferior face where the other veins are also slightly seen [20,21,22].

Inflorescence of these species are simple thyrse type. *S. adstringens* has snowy to yellowish inflorescences, rarely pinkish, geminate to ternate spikes, sometimes isolated, with 10–11 cm of length; *S. polyphyllum* has them in pink to reddish color, generally isolated spikes with 8–11 cm; while *S. rotundifolium* shows similar color of inflorescence as *S. adstringens* but 2–3 spikes of 9–18 cm of length [20]. Flowers are hermaphrodite (but *S. adstringens* and *S. rotundifolium* can rarely show male and female flowers), calyx and corolla are campanulate. *S. adstringens* has snowy to yellowish flowers, corolla of 5 mm of length; *S. polyphyllum* has reddish flowers, corolla of 3–3.5 mm and, *S. rotundifolium* presents snowy, light green to yellowish flowers also with 3–3.5 mm corolla [20,23]. They present seeds (8–10, 7–8 and 5–15, respectively in *S. adstringens*, *S. polyphyllum,* and *S. rotundifolium*) that are located in broad bean without salience [20,23]. Nucoide legume of *S. adstringens*is straight, with rounded apex and base, *S. polyphyllum* and *S. rotundifolium* have it straight but it is also rarely found slightly curved [20]. There are no significant macro- and microscopic differences between these species’ barks, which are commonly sold in free markets and therefore other assays could be done for identification, as tannin content [21,24].

Attempts of conservation and domestication of these species in order to keep their chemical composition and biological properties include: germination [25,26,27], micropropagation [28,29], callus culture [30,31], and genetic studies [32,33]. That is important since agricultural expansion eliminates native specimens and is an ecological concern that compels the sustainable management of these trees [34]. Even more, barks are extracted in a disorderly way from the trees for medicinal purposes and this exploitation reduces the regeneration process and the density of specimens, corroborating need for sustainable management, domestication, and conservation of the species [35,36,37].

## 5. Ethnopharmacological Uses

Traditional medicine is an important source of medicinal information, and over time humans have learned from Nature which plants, animals, and other elements could be used to help their survival. The wide biodiversity of flora in Brazil has provided an abundance of ethnopharmacologically important plants, and the use of “barbatimão” is consistently reported. It is noteworthy that the reports of the utilization of medicinal plants, including “barbatimão”, occurs mainly among adults with limited access to health care and is related to familiar traditional uses in addition to vendors in public markets or descendant of indigenous and maroon people [8,24,38,39,40].

Almost all collected papers about ethnopharmacological uses of “barbatimão” reported the treatment of wounds, followed by infections. Treatments for female genitourinary conditions and uterine disorders, and gastric ulcers were also frequently reported. Treatment of cancer was addressed in some ethnopharmacological surveys, as well as treatments of hemorrhage, diabetes, and pain. The most common preparations were infusions, macerations, and decoctions of the barks. *S. adstringens* and *S. rotundifolium* were the species most often observed. Cicatrizing, astringent, anti-inflammatory, and antimicrobial properties of the barks were the main attributes that characterized the folk choice of “barbatimão” and they were demonstrated scientifically [9,41,42]. Oral and topical were mentioned as the preferred routes of administration (Table 1).

One of the Brazilian policies related to the development of more economically accessible health treatments has been to stimulate the phytotherapy in the primary healthcare system [53], and “barbatimão” was one of the ethnopharmacologically-used plants included in the list of medicinal plants in the system [8]. Owing to this phytotherapeutic policy and the extensive ethnopharmacological use, the quality control, correct identification [54,55,56] and conservation of the species are essential requirements to ensure accurate treatment, to avoid toxicity or dose dilution with impurities [43]. The Brazilian Pharmacopeia includes one monograph for correct identification of *S. adstringens* barks, purity and dose assays based in its high tannin content [17], which can vary throughout the year in the different species [57,58]. Another ongoing concern is agricultural advance into habitat areas of these species [44].

Besides the stimulus for the national phytotherapeutic use of medicinal plants, ethnopharmacology has a cultural value and shelters an important source of molecules with biological activities and those are good reasons for more pharmacological studies and plant conservation practices.

## 6. Chemical Composition

### 6.1. Metabolites Identified in Stryphnodendron Species

**Figure 2:**
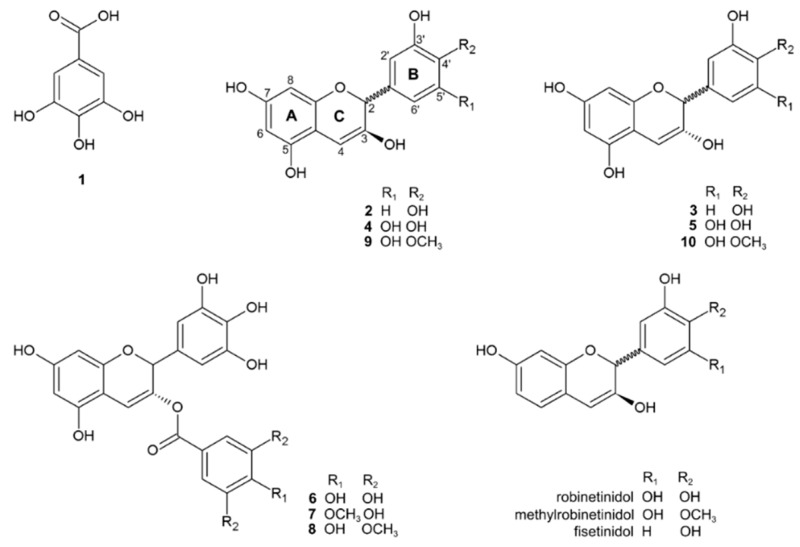
General chemical structure of gallic acid and proanthocyanidins identified in the bark of *Stryphnodendron* spp. known as “barbatimão”. Numbers indicate the respective molecule in Table 2, with respective substituent (R). Dimers and the polymer identified are repetition of these monomers according to their names in the corresponding carbon positions.

The stem bark of “barbatimão” is the main utilized part of the plant and, therefore, its chemical composition was extensively studied focusing on its secondary metabolites, which led to the identification of high phenolic or tannic contents and elucidation of the main low- or high-weight tannins present in aqueous, hydroalcoholic, and acetone:water extracts [59,60,61,62]. Some extracts of “barbatimão” were also prepared using propylene glycol and water as solvent for use in pharmaceutical formulations. In those cases, a higher content of tannins was obtained with 80% propylene glycol [63]. Chromatographic methods are mainly used for the isolation and identification of the presence of a variety of polyphenolic compounds, especially hydrolysable and condensed (as the proanthocyanidins, prodelphinidins, prorobinetinidins, and profisetenidins) tannins in extracts and fractions of “barbatimão” [64]. The compounds identified in the barks are presented in Table 2. The basic skeletal structure of hydrolysable tannins and proanthocyanidins are illustrated in Figure 2.

Nevertheless, polar extract of the leaves of *S. rotundifolium* also showed a high polyphenol content but not as high as that found in the bark. Gallic acid, catechin, caffeic acid, and rutin are examples of polyphenols identified in the leaves [60]. Previously, prodelphinidins, gallic acid, and flavonols were also described in the leaves of *S. adstringens* and *S. polyphyllum* [62], as well as saponins and cumarins [71]. The tannins in the leaves were identified as gallic acid, epicatechin-(4β→8)-catechin, and epicatechin 3-*O*-gallate [59]. Moreover, galactomannans were extracted from the seeds of *S. adstringens* [72].

Lopes et al. [54] observed that *S. adstringens* and *S. rotundifolium* differ by the presence of epigallocatechin 3-*O*-(3,5-dimethyl)gallate and epigallocatechin-3-*O*-(3-methoxy-4-hydroxy)-benzoate and are chemically related as they both contain the 5-deoxyproanthocyanidins-B-ring trihydroxylated (prodelphinidins), whereas *S. polyphyllum* shows the presence of the dihydroxylated form (profisetenidins). However, *S. adstringens* is the most studied “barbatimão” species and, so, *S. polyphyllum* and *S. rotundifolium* tannin composition descriptions may be incomplete.

Therefore, the chemical composition of the *Stryphnodendron* spp. includes a wide variety of tannins particularly of the condensed class with diverse forms, and it was possible to identify the proanthocyanidins as monomers, dimers, and polymers (Table 2). Those molecules have interesting properties and interactions with protein among other organic compounds, which is the basis of the biological activities of the bark [73].

### 6.2. Extraction and Analysis of Tannin Metabolites of Stryphnodendron Species

Because of their polarity, the most commonly described solvent for extracting tannins from the bark of *Stryphnodendron* spp. is the polar system consisting of acetone:water at a 7:3 (*v*/*v*) ratio [42,54,62,66,67,68,69,70]. Subsequently, the extracts were dried, partitioned with water and ethyl acetate, and then the ethyl acetate fraction was subjected to column chromatography (CC) using Sephadex LH-20. The subfractions generated by CC were chromatographically analyzed using multi-layer coil counter current chromatography (MLCCC), high-pressure liquid chromatography (HPLC), or CC until purification and identification of the tannins.

Thus, gallic acid was obtained using HPLC. The condensed tannin 4′-*O*-methylgallocatechin was separated using HPLC from an MLCCC (ethyl acetate:*n*-propanol:water, 140:8:80, *v*/*v*/*v*) subfraction using an isocratic methanol:acetonitrile:water (15:5:80, *v*/*v*/*v*) system, and identified using electron ionization mass spectrometry (EI-MS) and the acetylated form was identified using proton nuclear magnetic resonance (^1^H-NMR) [68]. Gallocatechin and epigallocatechin were separated after a second MLCCC, by HPLC (same isocratic system). Epigallocatechin 3-*O*-gallate in the same subfraction from the second MLCCC was purified using CC [68]. Purification of epigallocatechin 3-*O*-(3,5-dimethyl)gallate and epigallocatechin 3-*O*-(3-methoxy-4-hydroxy)benzoate required subjecting the subfraction obtained using the multi-layer method to MLCCC, generating a subfraction that was submitted to CC, followed by HPLC (using the same isocratic methanol:acetonitrile:water system), from which the compounds in the subfraction were acetylated, semi-purified using thin layer chromatography (TLC). Furthermore, a new HPLC procedure with a normal phase column (hexane:ethyl acetate, 55:45, *v*/*v*) provided the best separation of the compounds [68]. Epigallocatechin-(4β→8)-gallocatechin and epigallocatechin-(4β→8)-epigallocatechin were purified from the subfractions where they were concentrated using MLCCC and isocratic HPLC. However, epigallocatechin-(4β→8)-epigallocatechin-3-*O*-gallate was separated using MLCCC and HPLC with an isocratic system of methanol:water (3:1, *v*/*v*) [68]. In comparison, purification of epigallocatechin-(4β→8)-epigallocatechin 3-*O*-(4-hydroxy)benzoate was slightly different, using HPLC with an isocratic system of methanol:water at 21:79 (*v*/*v*) ratio [68]. In contrast, subfractions containing epigallocatechin 3-*O*-gallate-(4β→8)-epigallocatechin 3-*O*-gallate and epigallocatechin-(4β→6)-epigallocatechin were subjected to MLCCC and HPLC using a gradient system of methanol:water at 25–30%. Gallocatechin-(4β→8)-epigallocatechin 3-*O*-gallate and gallocatechin-(4α→8)-epigallo-catechin 3-*O*-(4-hydroxy)benzoate were purified from the subfrations following the same strategy, except that the gradient used for the HPLC were 24–27% and 28–30%, respectively [68]. Compounds were identified using EI-MS and ^1^H-NMR [68].

Prorobinetinidins were further purified from the ethyl acetate fraction by first subjecting it to CC using Sephadex LH-20, and then main subfractions were subjected to MLCCC (ethyl acetate:*n*-propanol:water, 35:2:2, *v*/*v*) and HPLC (reverse-phase C18). Therefore, robinetinidol-(4β→8)-epigallocatechin and robinetinidol-(4α→8)-gallocatechin were purified using HPLC and a methanol:acetonitrile:water (3:1:16, *v*/*v*/*v*) system, while robinetinidol-(4α→8)-epigallocatechin was purified using a methanol:water (21:79, *v*/*v*) system. The other prorobinetinidins were purified using a gradient system of methanol:water at 28–30% (robinetinidol-[4β→6(8)]-gallocatechin and robinetinidol-(4β→8)-epigallocatechin-3-*O*-gallate) and 25–30% (robinetinidol-(4α→6)-gallocatechin, robinetinidol-(4α→6)-epigallocatechin, and robinetinidol-(4α→8)-epigallocatechin-3-*O*-gallate) [69]. Subjecting different subfractions to MLCCC allowed the purification of 4′-*O*-methylrobinetinidol-(4α→8)-4′-*O*-methylepigallocatehin, 4′-*O*-methylgallocatechin, 4′-*O*-methylrobinetinidol-(4α→8)-4′-*O*-methylgallocatehin, gallocatechin, and epigallocatechin [54].Purification of the dimer 4′-*O*-methylgallocatechin-(4α→8)-4′-*O*-methylgallocatechin followed the same purification path: after extraction with acetone:water, the subfraction was partition using CC with ethyl acetate and water, and one of the subfractions from the MLCCC process produced the molecule that was identified by chemical shifts using ^1^H-NMR and ^13^C-NMR [54,70].

Metabolites from *S. polyphyllum* were similarly separated and analyzed, and after the extraction, partitioning, and CC, gallic acid was identified and the subfractions were subjected to other chromatographic purification procedures. Subsequently, 4′-*O*-methylrobinetinidol-(4β→6)-4′-*O*-methylgallocatechin was obtained from a second CC process using Sephadex LH-20 and gallocatechin, epigallocatechin, and 4′-*O*-methylgallocatechin were obtained from different subfractions following the MLCCC. A reverse and a normal-phase HPLC were used to obtain epigallocatechin-(4β→8)-gallocatechin. After the MLCCC procedure, the subfractions were subjected to a normal-phase column HPLC process, which produced profisetenidins fisetinidol-(4β→8)-gallocatechin and fisetinidol-(4β→8)-gallocatechin [54].

The water fraction from the partitioning step was subjected to CC using a Sephadex LH-20 column, which generated a subfraction rich in a polymer, identified using electrospray ionization (ESI)-MS and ^13^C-NMR, as a 2114 Da polymer with six monomers of flavan-3-ols and one galoil group consisting of prodelphinidin and prorobinetinidin units [42].

After identifying the purified compounds from the barks of *Stryphnodendron* species, the presence of the compounds was evaluated in studies using straight strategies. HPLC with reverse-phase column was used to analyze the organic phase of the fraction obtain from the liquid-liquid partitioning of ethanolic extracts from the barks of *S. adstringens* and *S. rotundifolium* using ethyl acetate:butanol:*i*-propanol:water (3.5:0.5:1.0:4.5, *v*/*v*/*v*/*v*), which was characterized by the presence of gallic acid, gallocatechin, epigallocatechin, catechin, and epigallocatechin 3-*O*-gallate [56,65]. The ethanolic extract of *S. adstringens* bark, which was partitioned with ethanol:*i*-propanol:*n*-butanol (42:12:6, *v*/*v*/*v*) and analyzed using ultraperformance liquid chromatography (UPLC) coupled to ESI-MS, was chemically characterized by the presence of gallic acid, gallocatechin, epigallocatechin, methylepigallocatechin 3-*O*-gallate, and the dimers of epigallocatechin, epigallocatechin 3-*O*-gallate, methylepigallocatechin3-*O*-gallate and epigallocatechin 3-*O*-gallate, epigallocatechin and epigallocatechin 3-*O*-gallate, 4′-*O*-methylgallocatechin and, methylepigallocatechin and epigallocatechin [64]. Analysis of the extract of *S. adstringens* obtained with acetone:water showed the characteristic chemical composition when subjected to MS and tandem MS (MS/MS): gallic acid, robinetinidol, epigallocatechin, 4′-*O*-methylepigallocatechin, epigallocatechin 3-*O*-gallate, epigallo-catechin-*O*-methylgallate, robinetinidol-epigallocatechin, robinetinidol-4′-*O*-methylepigallocatechin, epigallocatechin-epigallocatechin, 4′-*O*-methylepigallocatechin-4′-*O*-methylepigallocatechin, and epigallocatechin-epigallocatechin 3-*O*-gallate.

As summarized here, the purification of condensed tannins from *Stryphnodendron* was a particularly laborious task, involving several fractionations using suitable chromatographic methods. However, the chemical fingerprint of the bark can currently be obtained in a straightforward manner. To confirm the identity of “barbatimão” species, the Brazilian Pharmacopeia refers to the extract preparation with the acetone: water mixture and the characteristic presence of gallic acid, epigallocatechin, and 4′-*O*-methylgallocatechin [17].

## 7. Correlated Biological Activities

According to previous reports, the efficacy of the main ethnopharmacological uses of the bark of “barbatimão” were confirmed for the treatment of wounds, gastric wounds, and infectious and inflammatory disorders. The activity of the bark extract on other disorders such as cancer, pain, diabetes, blood pressure, and as a diuretic were evaluated but the results obtained were insufficient to scientifically prove its usefulness for these indications. Other activities not correlated to the ethnopharmacological applications such as anti-protozoal and antiviral (not against influenza), were also analyzed with promising preliminary results. For those activities, improved, established methods identified the compounds present in the plant sample tested, which could be associated with the confirmed activity (Table 3), paying more attention to the epigallocatechin 3-*O*-gallate and proanthocyanidin polymer of 2114 Da. A general description of the biological activities tested for in “barbatimão” from the *Stryphnodendron* genus is presented as follows.

### 7.1. “Barbatimão” Bark Promotes Wound Healing

The most common ethnopharmacological use stated for “barbatimão” stem bark is wound healing. Studies on animals and humans using only the extract or a pharmaceutical formulation in which it was incorporated, confirmed this biological activity. One of the earliest studies reported the wound healing activity of *S. adstringens* bark decoction at 1% on incisions in mice [74], which was also reported in similar studies performed subsequently [75,76,77]. Then, ointments containing the “barbatimão” bark extract were prepared and the wound healing activity was analyzed in rats and in humans. An ointment containing 10% of the aqueous bark extract was used to treat cutaneous wounds on rats and showed complete epithelization after 14 days with better inflammation and neovascularization process recovery than that of the control group treated with a physiological solution [78]. Subsequently, a 3% aqueous extract showed potential angiogenic activity, corroborating the previously observed neovascularization, which is an important step in the wound healing process [79,80]. Another ointment with 3% of the extract also healed patients with cutaneous wounds of decubitus position [77,81]. Based on those results, a topical preparation was developed with a composition of 1–6% total phenols from a dry extract of *S. adstringens* or *S. polyphyllum* and a patent was administered to the preparation [82]. An ointment containing 1% of a lyophilized ethyl acetate fraction (obtained from an acetone:water extract) of the bark of *S. adstringens* was similarly tested against wounds in rats, and its topical application stimulated epithelization [83].

In addition to reepithelization, the production of collagen fibers was also observed in wounds of diabetic rats treated with a gel formulation with a 1% acetone:water extract [66], and similar results were obtained with a 10% glycolic extract [84]. Further characterization of the acetone:water extract of the bark of *S. adstringens* indicated it contained almost 40% total polyphenol content and hydrolysable tannin gallic acid, prorobinetinidins robinetinidol, robinetinidol-epigallocatechin, robinetinidol-4′-*O*-methylepigallocatechin, prodelphinidin epigallocatechin, epigallocatechin 3-*O*-gallate, 4′-*O*-methyl-epigallocatechin, epigallocatechin-*O*-methylgallate, epigallocatechin-epigallo-catechin, 4′-*O*-methyl-epigallocatechin-4′-*O*-methyl-epigallocatechin, and epigallocatechin-epigallo-catechin 3-*O*-gallate [66]. It is interesting to note that the reepithelialization and angiogenesis effects have been described for epigallocatechin 3-*O*-gallate, which is one of the characteristic tannin metabolite in *Strpynhodendron* acetone:water bark extract [85,86].

Ointments containing 2.5% lyophilized acetonic extract or the ethyl acetate fraction from the barks of *S. rotundifolium* and *S. polyphyllum* were also tested on rat wounds and were shown to induce reepithelization. However, the epidermal growth was faster with the extract of *S. polyphyllum* and the ethyl acetate fraction of *S. rotundifolium* than with the other fractions. In that case, the first extract contained approximately 50% total phenolic content and 25% tannin content while the second fraction contained 89% and 36% phenolic and tannin contents, respectively [87]. The authors suggested that the differences were likely attributable to the presence of a variety of prodelphinidins, prorobinetinidins, and profisetenidins in *S. polyphyllum* although higher phenolic and tannin contents were observed in *S. rotundifolium*, whereas only prodelphinidins were observed in this last species. Prodelphinidins, prorobinetinidins, and a polymer of these two condensed tannins were also described in the bark of *S. adstringens*, indicating the diversity of its tannin composition that significantly corroborated the wound healing activity reviewed here. Therefore, the diverse condensed tannin contents could be more useful for wound healing than the content of a few structures, as was also observed for *S. adstringens*.

Treatment of gastric ulcer wounds with the bark of “barbatimão” has been widely reported as well and this application has been demonstrated using animal models (Table 3). Lesions induced by acute stress or acidified ethanol were significantly reduced after treatment with aqueous and butanolic fractions (obtained from an acetonic bark extract) of *S. adstringens* [88]. These observations indicate a concentration of specific polyphenols since the proanthocyanidins previously described were identified in the extract with wound healing activity. Another study showed the reduction of ulcer lesions induced by ethanol and hypothermic restraint-stress in rats pre-treated with an acetonic fraction (obtained from a methanolic extract) from *S. adstringens* [89]. The fraction also decreased the gastric secretory volume and elevated pH, showing an antisecretory effect [89]. However, both studies reported that the fraction did not have activity on indomethacin- or acetic acid-induced ulcers that could be caused by the indomethacin-induced inhibition of prostaglandin biosynthesis and not a potent antisecretory activity by the extract, as observed with cimetidine [88,89]. Tannins interact with proteins and, therefore, it has been proposed that the complex forms a protective layer for the recovery of the stomach endothelium and inhibition of the H^+^/K^+^ ATPase by hydrolysable tannin has also been observed, which reduces acid secretion and supports the results of *S. adstringens* on gastric ulcer treatment [90,91,92]. Although impressive results have been reported, the scientific studies of the internal use of these extracts against gastric ulcer were performed in rat models. Furthermore, one study reported a toxic effect that was comprehensively characterized [88], and should be considered. 

### 7.2. Anti-inflammatory Activity of “barbatimão” and Correlation to Antinociception

Another claimed ethnopharmacological property of the barks of these species is anti-inflammation. The acetonic fraction of *S. adstringens* inhibited rat paw edema with reduction of the exudate volume and migration of leukocytes. In addition, inhibition of paw edema was observed in a model with induced arthritis and decreased vascular permeability mediated by intraperitoneal administration of acetic acid in mice [93]. Antiedema results were similarly observed with a 1% solution of *S. adstringens* [94]. The aqueous and organic (ethanol:isopropanol:butanol) fractions (obtained from an ethanolic bark extract) of *S. adstringens* reduced the accumulation of neutrophils in the joint cavity of rats with arthritis induced by lipopolysaccharide (LPS) but this effect was not mediated by a decrease in C-X-C motif chemokine ligand 1 (CXCL1), the major chemokine recruiter of neutrophils [64]. The same study showed that tumor necrosis factor (TNF)-α production decreased in human monocytes THP-1 cells stimulated with LPS, which supports the anti-inflammatory claim for *S. adstringens* [64]. The organic fraction reduced neutrophil accumulation considerably more than the dexamethasone control did. Phytochemical evaluation of the organic fraction identified the presence of gallic acid and 11 different monomers and dimers of prodelphinidins, including epigallocatechin 3-*O*-gallate [64]. The attenuation of inflammatory process by some of those tannins, especially to epigallocatechin 3-*O*-gallate, was previously described [95,96,97], indicating a correlation between the anti-inflammatory activity of the stem bark of *S. adstringens* and the prodelphinidin constituents.

The use of “barbatimão” against pain could be related to its anti-inflammatory property. One study evaluated the aqueous and ethyl acetate fractions of the acetonic extract of *S. adstringens* against three pain models but an antinociceptive effect was only observed in the acetic acid- and formalin-induced writhing models [98]. It is interesting to note that the extract and aqueous fraction reduced the number of writhing compared to the saline control in two models of pain induced by inflammatory process. Specifically, 1) acetic acid induces capillary permeability, liberating substances that cause pain in nerve ends [99] and 2) the late phase of the formalin test, which is mediated by an inflammatory process, is reported as a return of the nociception minutes after formalin injection, and is used to elucidate pain and analgesia mechanisms [100,101]. The aqueous fraction contained concentrated levels of the proanthocyanidin polymer of 2114 Da, whereas the dimers where found in the ethyl acetate fraction, indicating that the peripheral antinociceptive effect of *S. adstringens* is partly due to the polymer of the condensed tannin [98]. Interestingly, epigallocatechin 3-*O*-gallate was also associated with antinociception in bone cancer due to the attenuation of inflammation by the reduction of TNF-α expression [96].

### 7.3. Antioxidant Property Might Mediate Claimed Anticancer Activity

The reactive oxygen species (ROS) scavenging activity of the bark extract of *S. adstringens* prepared with 50% and 70% ethanol, acetone:water (7:3, *v*/*v*), and chloroform was comparatively evaluated based on the reduction of the reagent 1,1-diphenyl-2-picrylhydrazyl (DPPH) to DPPHH. Polar extracts showed scavenging capacity as high (95%) as that of the controls rutin (97%), gallic acid (97%), and vitamin C (98%) at the same concentration. However, the chloroformic extract, which showed irrelevant levels of total phenolic content, exhibited <75% scavenging capacity. TLC plates stained with DPPH presented a spot representing reduction at same retention time as that of the tannin spots [102]. The scavenging capacity of the acetonic extract, aqueous and ethyl acetate fractions, and CC subfractions of the stem bark of *S. rotundifolium* was similarly evaluated using TLC stained with DPPH and all samples reduced the free radicals [103]. Subsequently, the DPPH scavenging potential of hydroalcoholic bark extracts and aqueous extracts of the barks and leaves of *S. rotundifolium* was measured and both bark extracts showed better activity than that of vitamin C, whereas the leaf extract showed comparable scavenging capacity to that of the control at higher concentrations [60]. According to the authors, the bark extract contents of gallic acid, catechin, caffeic acid, and rutin were higher than those in the leaf extract, which explained the different antioxidant activity of the samples. 

Comparison of the acetonic extract and ethyl acetate fraction of *S. rotundifolium* and *S. polyphyllum* revealed the greater scavenger capacity of the second species, similar to the results of the wound healing potential comparison [87]. Therefore, the proanthocyanidin constituent of the barks of *Stryphnodendron* spp. and the potent scavenging capacity of the extracts, justify their ethnopharmacological use as anticancer agent; however, scientific studies need to be performed to prove that application and establish the best extract, compound, and concentration for use.

Furthermore, an ethyl acetate fraction obtained from the acetone:water extract of the leaves of *S. adstringens* was evaluated for antioxidant and anticancer potential. It showed good antioxidant activity by reducing iron, inhibiting protein oxidation and scavenging DPPH at same 50% radical inhibition concentration as vitamin C. The fraction did not show cytotoxicity for non-cancerous rat primary bone marrow but was cytotoxic to MCF-7 and MDA-MB-435 human breast cancer cell lines, and altered their morphology in addition to inducing DNA cleavage, apoptosis, and autophagy [59]. A tannin, epigallocatechin 3-*O*-gallate, present in the leaf fraction has already been designated as an anticarcinogenic compound [104]. The oxidation of epigallocatechin 3-*O*-gallate by O_2_^‒^ species was observed in the galloyl group, thereby modulating ROS production, which in combination with the inhibition of nuclear factor-κB, activation of mitogen-activated kinases, and inhibition of DNA methyltransferases, suggests prodelphinidin is a strong anticancer agent [104,105]. *S. adstringens* saline extract also reduced the technetium-99 m (used in nuclear medicine) labeling of red blood cells probably because of the redox or chelating properties of tannins [106]. 

### 7.4. Antimicrobial Activity Corroborates Oral and Genitourinary Use

It is notable that the ethnopharmacological use of *Stryphnodendron* spp. for infection treatment has been scientifically demonstrated mostly against gram-positive bacteria and pathogenic yeasts, as described in Table 4.

Interestingly, the hydroalcoholic extract (containing tannin pyrogallates, flavones, flavonols, xanthones, chalcones, aurones, flavononols, and flavonones) showed synergistic activity with the antibiotics gentamicin, kanamycin, amikacin, and neomycin against *Escherichia coli* and *S. aureus* [118]. In contrast, a saline extract of the seeds of *S. rotundifolium* did not inhibit *S. aureus*, *E. coli*, and *Pseudomonas aeruginosa* [119]. Hence, the antibacterial activity of gallic acid and different proanthocyanidins [120,121] could justify the use of the stem bark of *Stryphnodendron* spp. in disorders as sore throat and dental and urinary infections.

On the other hand, the antifungal activity of the “barbatimão” species is more significant and different studies have attempt to elucidate the mechanism involved in the *Candida* spp virulence, biofilm inhibition, as well as the main active compound [42,114,122]. The propylene glycol extract of *S. adstringens* inhibited the formation of *C. albicans* biofilm on acrylic resins [123]. To elucidate the main active compound, Ishida et al. [42] performed bioguided extract fractionation according to the growth inhibition of *C. albicans*, which yielded a group of subfractions from the aqueous partition and the ethyl acetate fraction contained the polymer of 2114 Da with six units of flavan-3-ol and one galloyl group. This subfraction had similar inhibitory activity to that of nystatin, but showed fungistatic effect. Moreover, it decreased the *Candida* spp. virulence effect of adherence and filamentous formation in *C. albicans*, and enhanced the yeast phagocytosis by macrophage. A similar activity was reported for the polymer-rich subfraction against *C. tropicalis*, and biofilm reduction was also observed following pretreatment of planktonic cells with the subfraction or treatment during the adherence and matrix formation [114]. Further, the subfraction decreased the metabolic activity of sessile and dispersed cells during biofilm formation and maturation, indicating that the subfraction penetrated the biofilm matrix [124]. These findings are very important to developing strategies for avoiding the contamination of implanted medical devices and dissemination of the infection by dispersion cells [125].

Genitourinary disorders, especially in women, are among the most ethnopharmacological uses of “barbatimão”, since the widespread and recurrent vulvoginal candidiasis infection [126] can be treated with the stem bark of *Stryphnodendron* spp. Furthermore, the proven activity of the stem bark against *Candida* could contribute to the standardization of the extract and pharmaceutical formulations to effectively combat the pathogen.

In addition, the polymer-rich fraction inhibited the growth of *Cryptococcus neoformans* despite its fungistatic effect [115] and the hexanic leaf extract of *S. adstringens* inhibited the growth of different clinical isolates of the dermatophyte *Trychophyton rubrum* [116]. These findings could explain the folkloric use of this fraction for itching, chilblain, and dermatitis and is extremely important since both fungi present concerns of resistance to antifungals [127,128].

The antimicrobial activities of “barbatimão” could be related to the activity of the tannin content. Scalbert [129] proposed three different tannin antimicrobial mechanisms of action. These are inhibition of enzymes of the microorganism (correlating with protein interaction); microorganism deprivation of nutrients such as metals (explained by the complexation with tannin hydroxyl groups), and inhibition of oxidative phosphorylation (due to the antioxidant property). In addition, it is noteworthy that the compound epigallocatechin 3-*O*-gallate also exhibits antimicrobial activity. It has shown antibacterial (mainly against gram-positive bacteria), antibiofilm, and anticandidal activities, which enhances the understanding of the *Stryphnodendron* species antimicrobial activity [130].

### 7.5. Effect on Diabetes, Blood Pressure, and Diuresis

This review found only one study on the use of *Stryphnodendron* spp. in diabetes. Low concentrations (1.86 and 0.61 µg/mL) of the ethanolic extract of the bark of *S. adstringens* inhibited the enzymes α-amylase and α-glucosidase, which indicates its potential blood glucose-lowering effects [131]. Moreover, the enzymatic inhibition likely corresponds to the general interaction of tannins with proteins, but a more specific interaction with the active site could also be possible [73,131]. Further studies on diabetes would be important since epigallocatechin 3-*O*-gallate also improved the insulin sensitivity of rats and myocytes [132]. Another study [107] reported the reduction of blood pressure in dogs with normal arterial pressure following treatment with 8.2–12.3 mg/kg of acetone:water extract of the bark of *S. adstringens* and the fractions obtained with ethyl acetate, butanol, and water.

Analysis of the bark of *S. adstringens* and *S. rotundifolium* as dry powder on water renal excretion in mice revealed an antidiuretic effect, whereas the dry powder of the seeds of *S. rotundifolium* induced the claimed diuretic effect with no electrolyte excretion changes [133,134].

However, the data of the activities described in this section are currently inconclusive and further detailed analysis must be performed to ensure the proper use of extracts in the phytotherapy of diabetes, hypertension, and for diuresis. The evaluation of the toxicity of these extracts is an important topic that needs to be summarized, particularly with the allegedabortive effects of the seeds, which are described later.

### 7.6. Anthelmintic Activity and Other Promising Activities

The claimed folkloric anthelmintic activity of the bark was tested against *Schistosoma mansoni* using the acetone:water extracts of *S. adstringens* and *S. polyphyllum*, which both showed a faster larvicidal activity against miracidia and cercariae forms than the control treatment did [135]. This finding supports the validity of this reported application of the extracts. In addition, extracts of the barks and leaves of *S. adstringens* and *S. polyphyllum* showed molluscicidal activity against *Biomphalaria glabrata*, the snail intermediate host of *S. mansoni* [136,137,138].

Studies of the ctivity of *Stryphnodendron* spp. against protozoans commenced when the acetonic extract of the bark of *S. adstringens* was shown to inhibit the cell growth of *Herpetomonas samuelpessoai*. It is a non-pathogenic trypanosomatid used as an experimental model because it shares antigens with *Trypanosoma cruzi*, causing immune response and is susceptible to the same drugs used to treat *T. cruzi* [139]. Subsequently, inhibitory concentrations of the acetonic extract as well as the aqueous and ethyl acetate fractions were determined and ultrastructural alterations in cells were observed using transmission electron microscopy, in addition to decreased activity of the mitochondrial enzyme succinate cytochrome c reductase [140]. The extract of *S adstringens* (100 µg/mL) showed weak growth inhibition of the pathogenic protozoan *Leishmania amazonensis* in the promastigote and amastigote form and moderate inhibition of *T. cruzi* in the epimastigote form [141]. In an in vivo study, the ethanolic extract decreased the number of f *T. cruzi* parasites in the blood of inoculated mice [142]. The ethanolic extracts of the barks of *S. adstringens* and *S. polyphyllum* also exhibited trypanocidal activity, reducing the number of parasites in infected mice faster than the control group mice [143].

Then, an ethanolic extract of the bark of *S. rotundifolium* and its aqueous and organic fractions were tested against promastigote forms of *L. amazonensis*, which they highly inhibited and this effect was potent with the tannins isolated from the organic phase. Gallic acid exhibited the best inhibition (50% of promastigote inhibition at 1.7 µg/mL), followed by the epigallocatechin 3-*O*-gallate [65]. The hydroalcoholic extract of *S. rotundifolium* was also tested against *L. brasiliensis* and *L. infantum* promastigotes and *T. cruzi* epimastigotes and the mortality rates at 1000 µg/mL were of 56, 45, and 82%, respectively [144]. This observation indicates that the best activity of *S. rotundifolium* was achieved with the organic fraction and gallic acid was the most active compound against *L. amazonensis*. These results show the promising antiprotozoal activity of *Stryphnodendron* species, especially their tannin content, and further analysis should be carried out to determine the inhibitory agents, appropriate concentrations, and in vivo activity.

Ethnopharmacology data indicates the use of the stem bark of *S. rotundifolium* against influenza and the antiviral activity of *S. adstringens* bark was assayed using the aqueous and ethyl acetate fractions against poliovirus 1 (P-1) and bovine herpesvirus 1 (BHV-1) in HEp-2 cells. Both extracts inhibited the viral replication and the prodelphinidins catechin, epicatechin, gallocatechin, and epigallocatechin were identified in the ethyl acetate fraction [67].

Taking together, these results indicate the antiviral activity of the extract and its effective wound healing activity and, therefore, it was formulated and patented as an ointment with different “barbatimão” species for used against the human papillomavirus (HPV), particularly in the prevention of cervical cancer [145].

Another ointment was formulated using the aqueous extract of *S. adstringens*, and was found to reduce the hemorrhagic and myotoxic effects in mice caused by the venom of *Bothrops pauloensis*, and these effects were claimed to be mediated by an interaction between the tannins and the toxic proteins of the venom [146].

The bark and leaf extracts of *S. rotundifolium* and *S. adstringens* had little or no inhibitory effects on *Rhipicephalus microplus* (tick) [147], *Rhopalosiphummaidis* (corn aphid) [148] and *Diabrotica speciose* (the larva of this insect perforates potatoes) [149].

Therefore, the anthelmintic folkloric uses of this species has also been confirmed, but requires further detailed investigation. Among the activities not mentioned in ethnopharmacological surveys, the molluscicidal, antiviral, and antiprotozoal activity should receive more attention to confirm the promising results reported here to facilitate the standardization process for the use of the bark for these applications.

## 8. Cytotoxicity and Toxicology

As seen, stem bark of “barbatimão” from *Stryphnodendron* spp. has diverse uses in folk medicine and some of them were confirmed by scientific studies. Therefore, it is very important to ensure the safety of the active extracts and products obtained of the species in this genus.

In that sense, cytotoxic effects against mammalian cell lines is a recurrent property analyzed. The acetonic extract of the barks of *S. adstringens* did not cause hemolysis of sheep erythrocytes [141] neither did the aqueous and ethyl acetate fractions and subfractions. The same samples were not cytotoxic at 50% (50% cytotoxic concentration, CC_50_) of Vero and macrophages J774G8 cells up to 100 µg/mL and showed hemagglutinant effect above 500 µg/mL [42]. Likewise, the ethanolic extract, aqueous, and organic fractions of *S. rotundifolium* and the isolated compounds gallic acid, gallocatechin, epigallocatechin, catechin, and epigallocatechin 3-*O*-gallate showed CC_50_ against macrophages at 100-300 µg/mL and did not cause lysis of human red blood cells [65]; the hydroalcoholic extract of *S. rotundifolium* showed CC_50_ against fibroblast cells at 190.24 µg/mL [144]. According to all these reports, it is important to pay attention to the active concentration of samples to each biological activity and evaluate whether the cytotoxic concentration is smaller to indicate safe use. For example, ethyl acetate subfraction polymer-rich has anti-*Candida* activity bellow 10 µg/mL, whereas antibacterial and anti-protozoal activities are achieved close to 1000 µg/mL.

Cytotoxicity by genotoxic effects of ethanolic extracts of *S. adstringens* was evaluated but no DNA damage was observed, for instance, no mutagenicity activity was observed with the Ames test using *Salmonella typhimurium* strains [150], or by somatic mutation and recombination test or by chromosome damage in germ cells of *Drosophila melanogaster*, or even by mutation in larvae or in adult male of the same insect [151]. Otherwise, it was shown that aqueous extract and fraction and hydroalcoholic extract of the leaves of *S. adstringens* exhibited antigenotoxic effects by reducing DNA damage and micronuclei formation in bone marrow cells from rats treated with the genotoxic cyclophosphamide [152].

Acute toxicity and 50% lethal dose (LD_50_) are somewhat different among the extracts of *S. adstringens*. The LD_50_ of hydroalcoholic bark extract was considered high, at the concentration of 250 µg/mL, when administrated intraperitoneally in mice for 14 days [153]. An alcoholic extract of *S. adstringens* showed LD_50_ of 250 mg/kg also intraperitoneally in mice, did not show skin primary irritation or ocular alterations in rabbits [145]. No behavioral change was observed in rats receiving acetonic extract, and it did not show high LD_50_ (2699 mg/kg) though daily oral dose of 800 mg/kg for 30 days decreased the animals’ weight, promoted thymic involution and increased glucose and aspartate aminotransferase levels in their plasma [154]. Those effects could reflect alterations in cell metabolism and it was observed that the extract impaired mitochondrial oxidative phosphorylation and liver metabolism by increasing oxygen consumption [155]. In opposition, a methanolic extract did not show acute toxicity or histological alterations in liver and kidney of treated rats, neither showed hepatic or renal dysfunctions in rabbits [117].

However, when evaluating genotoxicity and acute toxicity of the polymer-rich subfraction obtained from the ethyl acetate fraction of the barks of *S. adstringens*, a safer sample was detected. In fact, it is possible to see a cytoprotective effect against mutagenicity, low cytotoxicity against *Artemia salina* [156], a LD_50_ higher than 3000 mg/kg in mice and chronic toxicity evaluation for 90 days with 200 mg/kg of the subfraction did not show biochemical, hematological, and histopathological effects [157]. Meanwhile, epigallocatechin 3-*O*-gallate caused hepatocyte disfunction and damage to mitochondria at high doses [158] and embryonic cytotoxicity [159].

Although broad beans of *Stryphnodendron* spp. are not used ethnopharmacologically, it is worth pointing out their abortive effect in animals. To confirm the information from farmers about cow abortion after consuming the beans from *S. rotundifolium*, a study administered 5 g/kg/day for 9 to 26 days. In fact, four out of seven cows had abortion and the behavior of others was abnormal, with decreased activity and appetite, salivation, difficulty in getting up, unstable gait, muscular tremors, and loss of weight [160]. Evaluation of toxic dose showed death of bovines with 60 g/kg of broad beans administered once and death from 10 g/kg on repeated days (for 8 days). Other diverse poisoning symptoms were also observed as well as diverse histopathological alterations [161,162]. Seed extracts of *S. adstringens* and *S. polyphyllum* showed abortive effect on female rats, corroborating to the precaution needed with the trees on farms with animals [11]. A congruent evaluation of cytotoxic and toxic effects and doses correlated with the different biological activities is still required for ensuring the safe use of the barks of “barbatimão” as well as the use of isolated compounds. No teratogenic or neurotoxic activity has been reported for the extracts in animals or humans.

## 9. Conclusions

Ethnoknowledge is still one of the most important tools for drug discovery, and phytotherapeutic treatments are easily accessible to the majority of the population. Ethnopharmacological uses of “barbatimão” from the genus *Stryphnodendron* genus were confirmed for different purposes, which would help guide the best approach for handling the stem bark extracts by the general population and would stimulate the development of pharmaceutical formulations. Topical use of the bark extract as a wound-healing agent, in order to take advantage of its antimicrobial action, is a well-established application of *Stryphnodendron* spp. Unfortunately, there is still insufficient scientific data about the correlation between dosage and pharmacological and toxicological parameters with regard to other activities, which can be explored as long as the mechanism of action of the bioactive compounds. A better understanding of those parameters could also motivate further studies on tannin from “barbatimão” aid the development of a drug that may be made available worldwide. It is also important to bear in mind the need to conserve these plants by adopting appropriate harvesting of the barks.

## Figures and Tables

**Table 1:** Overview of ethnopharmacological reports in literature about “barbatimão” uses.

Species	Part of Plant	Medicinal Use	Form of Preparation and Administration	Reference
SA	Stem bark	Uterine infection, ovary inflammation, wound healing, ulcer, cicatrizing, anti-inflammatory, hygiene, sore throat and itch	Baths	[43]
SA	Stem bark	Ulcerous wounds	Macerated, used as bath	[44]
SA	Stem bark	Not mentioned	Topical use	[45]
SA	Stem bark	Wound healing	Decoction, infusion or macerated, for external or internal uses	[8]
SA	Stem bark	Wound, chilblain, diabetes, prostate problems, inflammation, gastritis, liver diseases, dental inflammation, pain in general	Not mentioned	[39]
SA	Stem bark	Leucorrhea, wound healing, ulcer and vaginal discharge	Not mentioned	[46]
SA	Stem bark	Urinary infection	Oral	[47]
SA	Stem bark	Wound healing	Tea, infusion, bottleful, powder	[48]
SR	Stem bark and seeds	Diuretic, anti-diarrheic, ulcer, cicatrizing, chilblain, astringent, for gums	Macerated in water	[49]
SR	Stem bark	Wounds, inflammation, gastritis and ulcer, vaginal inflammation, pain, infection, prostate disorders, sexually transmitted diseases, rheumatism, hypertension, dermatitis, burns, menopause, postpartum healing, renal calculi, influenza, lung diseases	Immersion in water for oral or topical administration	[50]
SR	Stem bark, roots and leaves	Inflammation, vaginal discharge, urinary infection, uterine lesions	Decoction and infusion	[51]
SR	Stem bark	General wound healing, ulcer, general inflammation, headache, gastritis, cancer, fever, leg, body, stomach and belly pain, cough, cuts, scabs, flu, sore throat, heart, childbirth inflammation, blood pressure, blood disorder, kidneys, lung inflammation, sinus and urinary infection, excessive menstruation, itch, vaginal discharge, stanch blood from cuts, skin allergy, swelling, tightening the vagina for sexual intercourse	Not mentioned	[36]
SR	Stem bark and roots	Backache	Macerated for oral administration	[52]
SR	Stem bark	Wound, uterus and skin inflammation, wound healing, genital disease and cancer	Immersion in water or decoction is prepared for oral and topical administration and baths	[40]
SR	Stem bark	Ulcer, wound healing, venereal disease, hemorrhage, diabetes, anthelmintic, high blood pressure, anemia, cancer, liver disease	Infusion and tincture	[24]

SA: *S. adstringens*. SR: *S. rotundifolium*.

**Table 2:** Compounds identified in the stem bark of *Stryphnodendron* species known as “barbatimão”.

Number	Compound Name	Species	Reference
**1**	Gallic acid	SA, SP, SR	[54,56,60,62,64,65,66]
**2**	Catechin	SA, SR	[56,60,65,67]
**3**	Epicatechin	SA	[67]
**4**	Gallocatechin	SA, SP, SR	[54,56,62,64,65,67,68]
**5**	Epigallocatechin	SA, SP, SR	[54,56,62,64,65,66,67,68]
**6**	Epigallocatechin 3-*O*-gallate	SA, SR	[56,61,64,65,66,68]
**7**	Epigallocatechin 3-*O*-methylgallate	SA	[66]
**8**	Epigallocatechin 3-*O*-(3,5-dimethyl)gallate	SA	[54,68]
**9**	4′-*O*-methylgallocatechin	SA, SP	[54,64,68]
**10**	4′-*O*-methylepigallocatechin	SA	[66]
**11**	4′-*O*-methylepigallocatechin-3-*O*-gallate	SA	[64]
**12**	Epigallocatechin 3-*O*-(3-methoxy-4-hydroxy)benzoate	SA	[54,68]
**13**	Gallocatechin-(4α→8)-epigallocatechin 3-*O*-(4-hydroxy)benzoate	SA	[54,68]
**14**	Epigallocatechin-(4β→8)-epigallocatechin 3-*O*-(4-hydroxy)benzoate	SA	[54,68]
**15**	Gallocatechin-(4β→8)-epigallocatechin 3-*O*-gallate	SA	[68]
**16**	Epigallocatechin-(4β→8)-gallocatechin	SA, SP	[54,68]
**17**	Epigallocatechin-(4β→8)-epigallocatechin	SA	[64,66,68]
**18**	Epigallocatechin-(4β→6)-epigallocatechin	SA	[68]
**19**	Epigallocatechin-(4β→8)-epigallocatechin3-*O*-gallate	SA	[68]
**20**	Epigallocatechin 3-*O*-gallate-(4β→8)-epigallocatechin 3-*O*-gallate	SA	[64,68]
**21**	4′-*O*-methylepigallocatechin 3-*O*-gallate-epigallocatechin 3-*O*-gallate	SA	[64]
**22**	Epigallocatechin-epigallocatechin 3-*O*-gallate	SA	[64,66,68]
**23**	4′-*O*-methylepigallocatechin-epigallocatechin	SA	[64]
**24**	4′-*O*-methylepigallocatechin-4′-*O*-methylepigallocatechin	SA	[66]
**25**	Robinetinidol	SA	[66]
**26**	Robinetinidol-(4α→8)-epigallocatechin	SA	[66,69]
**27**	Robinetinidol-(4β→8)-epigallocatechin	SA	[69]
**28**	Robinetinidol-4′-*O*-methylepigallocatechin	SA	[66,69]
**29**	Robinetinidol-(4β→8)-epigallocatechin-3-*O*-gallate	SA	[69]
**30**	Robinetinidol-(4α→8)-epigallocatechin-3-*O*-gallate	SA	[69]
**31**	Robinetinidol-(4α→6)-gallocatechin	SA	[69]
**32**	Robinetinidol-(4α→6)-epigallocatechin	SA	[69]
**33**	Robinetinidol-[4β→6(8)]-gallocatechin	SA	[69]
**34**	Robinetinidol-(4α→8)-gallocatechin	SA	[69]
**35**	4′-*O*-methylrobinetinidol-(4α→8)-4′-*O*-methylgallocatechin	SA	[54]
**36**	4′-*O*-methylrobinetinidol-(4α→8)-4′-*O*-methylepigallocatechin	SA	[54]
**37**	4′-*O*-methylgallocatechin-(4α→8)-4′-*O*- methylgallocatechin	SA	[54,70]
**38**	4′-*O*-methylrobinetinidol-(4β→6)-4′-*O*-methylgallocatechin	SP	[54]
**39**	Fisetinidol-(4α→8)-gallocatechin	SP	[54]
**40**	Fisetinidol-(4β→8)-gallocatechin	SP	[54]
**41**	Polymer of 2114 Da of molecular weight with 6 monomers of flavan-3-ols and one galoil group consisting of prodelphinidin and prorobinetinidin units with configuration 2,3-*cis* and 2,3-*trans*	SA	[42]
**42**	Caffeic acid	SR	[60]
**43**	Rutin	SR	[60]

SA: *S. adstringens*. SP: *S. polyphyllum*. SR: *S. rotundifolium*.

**Table 3:** Ethnopharmacological uses scientifically studied and correlated compounds (identified by their numbers in Table 2).

Ethnopharmacological Use	Scientifically Observed?	Related Compound
Wound healing	Yes	1, 5–7, 9, 17, 22, 24–28
Gastric ulcer	Yes, but toxicity was observed	1, 5–7, 9, 17, 22, 24–28
Anti-inflammatory	Yes	1, 4–6, 9, 11, 17, 20–23
Against pain	Yes—peripheral antinociception	6 and 41
Cancer	Antioxidant activity has been extensively evaluated, but anticancer activity is not conclusive	1, 2, 6, 42 and 43
Antimicrobial-oral and genitourinary infections	Yes—against gram positive bacteria and *Candida* species	1 and 41

**Table 4:** Reports of antimicrobial activity of different extracts or fractions of "barbatimão".

Species	Extracts/Fraction	Microorganisms	Reference
SA	Hydroalcoholic and acetone:water extracts from the bark	*Staphylococcus aureus*	[41,102,107,108,109]
SA	Hydroalcoholic bark extract	*Staphylococcus epidermidis*; *Enterococcus faecalis*; *Streptococcus salivarius*; *Streptococcus sanguinis*; *Streptococcus mitis*; *Streptococcus mutans*; *Streptococcus sobrinus*; *Lactobacillus casei*	[41,102,110]
SA	Ethanolic and hexanic bark extracts	*Candida albicans*; *Streptococcus mutans*; *Aggregatibacter actinomycetemcomitans*	[111]
SA	Propylene glycol	*S. aureus*; *S. epidermidis*; *S.mutans*; *C. albicans*; *C. tropicalis*; *C. glabrata*	[112]
SA	Hydroalcoholic bark extract	*Mycobacterium tuberculosis*	[113]
SP	Ethyl acetate fraction	*S. aureus*; *Bacillus subtilis*	[87]
SR	Ethyl acetate fraction	*S. aureus*	[87]
SA	Polymer-rich subfraction	*C. albicans*; *C. tropicalis*, *Cryptococcus neoformans*	[42,114,115]
SA	Hexanic leaf extract	*Trychophyton rubrum*	[116]
SA	Methanolic extract and tannin fraction from the bark	*Pythium insidiosum*	[117]

SA: *S. adstringens*. SP: *S. polyphyllum*. SR: *S. rotundifolium*.
